# Intercellular cytosolic transfer correlates with mesenchymal stromal cell rescue of umbilical cord blood cell viability during *ex vivo* expansion

**DOI:** 10.3109/14653249.2012.697146

**Published:** 2012-07-10

**Authors:** Pat P. Y. Chu, Sudipto Bari, Xiubo Fan, Florence P. H. Gay, Justina M. L. Ang, Gigi N. C. Chiu, Sai K. Lim, William Y. K. Hwang

**Affiliations:** 1 Department of Hematology, Singapore General Hospital, Singapore; 2 Singapore Cord Blood Bank, Singapore; 3 Department of Clinical Research, Singapore General Hospital, Singapore; 4 Cancer and Stem Cell Biology, Duke-NUS Graduate Medical School, Singapore; 5 Department of Pharmacy, National University of Singapore, Singapore; 6 Institute of Medical Biology, A^*^ STAR, Singapore

**Keywords:** cell viability, intercellular transfer, mesenchymal stromal cells, umbilical cord blood

## Abstract

*Background aims*. Mesenchymal stromal cells (MSC) have been observed to participate in tissue repair and to have growth-promoting effects on *ex vivo* co-culture with other stem cells. *Methods*. In order to evaluate the mechanism of MSC support on *ex vivo* cultures, we performed co-culture of MSC with umbilical cord blood (UCB) mononuclear cells (MNC) (UCB-MNC). *Results*. Significant enhancement in cell growth correlating with cell viability was noted with MSC co-culture (defined by double-negative staining for Annexin-V and 7-AAD; *P*<0.01). This was associated with significant enhancement of mitochondrial membrane potential (*P*<0.01). We postulated that intercellular transfer of cytosolic substances between MSC and UCB-MNC could be one mechanism mediating the support. Using MSC endogenously expressing green fluorescent protein (GFP) or labeled with quantum dots (QD), we performed co-culture of UCB-MNC with these MSC. Transfer of these GFP and QD was observed from MSC to UCB-MNC as early as 24 h post co-culture. Transwell experiments revealed that direct contact between MSC and UCB-MNC was necessary for both transfer and viability support. UCB-MNC tightly adherent to the MSC layer exhibited the most optimal transfer and rescue of cell viability. DNA analysis of the viable, GFP transfer-positive UCB-MNC ruled out MSC transdifferentiation or MSC-UCB fusion. In addition, there was statistical correlation between higher levels of cytosolic transfer and enhanced UCB-MNC viability (*P*< 0.0001). *Conclusions*. Collectively, the data suggest that intercellular transfer of cytosolic materials could be one novel mechanism for preventing UCB cell death in MSC co-culture.

## Introduction

Mesenchymal stromal cells (MSC), also known as multipotent mesenchymal stem cells, have been used in various clinical applications such as tissue engineering (1), regenerative medicine (2) and immunoregulatory therapy (3). MSC can be isolated easily from various tissue sources (4), readily expanded in culture (5) and differentiated into various cell types, such as osteoblasts, chondrocytes and adipocytes, with suitable stimulation (6,7). Therefore, MSC have been proposed as a promising source of cells for site-specific repair of bone, cartilage, muscle, tendon, marrow stroma and other connective tissues (6). In addition, it has been shown that MSC have immunosuppressive and anti-inflammatory effects that are useful for the treatment of transplant-related complications such as graft-versus-host disease (GvHD) (8–10). Currently, many clinical trials are underway to explore the above-mentioned properties of MSC (11).

Despite the use of MSC in many clinical trials, the underlying mechanism by which it affects tissue repair and immunosuppression remains largely unknown. MSC have been postulated to differentiate into cells of the target tissue and functionally regenerate damaged, dysregulated or aged cells. In addition, direct and indirect effects of either cell – cell interaction or secreted, soluble factors may play a role in creating a local immunosuppressive environment (12). However, the above biologic effects of MSC still remain controversial (13) and may not represent the complete picture.

MSC have been found to enhance the viability of some cell types in co-culture systems (14–16). For the *ex vivo* expansion of hematopoietic stem (HSC) and progenitor cells (HPC), it has been suggested that the MSC layer used in the co-culture system could provide soluble factors such as cytokines/chemokines, extracellular matrix (ECM) proteins and adhesion molecules that serve to regulate survival and maintenance of ‘ stemness ’. One key consequence of using MSC co-culture for HSC expansion is the ability of HSC to bypass the need for CD34 selection prior to expansion (17,18). While several groups have demonstrated the utility of MSC in umbilical cord blood (UCB)-mononucleated cell (UCB-MNC) expansion, it remains to be determined whether this expansion is the result of increased cell proliferation or reduced apoptosis. As such, we have used a co-culture system of MSC with umbilical cord blood (UCB) cells as a model to investigate the mechanism by which MSC could support the *ex vivo* expansion of UCB-MNC.

Our data show that the viability supporting effect of MSC is most prominent when UCB-MNC and MSC are in physical contact, and results in reversal of early apoptosis and enhancement of the mitochon-drial membrane potential of the UCB-MNC. Furthermore, we show that there is transfer of cytosolic content from MSC to UCB-MNC, and this could contribute to the reversal of early apoptosis in the UCB-MNC cells. We propose that this mechanism may, at least partly, contribute to the effect of MSC observed in clinical studies where tissue viability is restored after injection or implantation.

## Methods

### Cell cultures

The human embryonic stem cell (ESC)-derived MSC (ES-MSC) HuES9.E1 cell line was generated according to the protocol published by Lian *et al.* (19) and was maintained on a gelatin-coated (0.1% w/v gelatin in Ultrapure H_2_O; Millipore, Billerica, MA, USA) tissue culture plate/flask surface (Becton Dickenson Falcon, San Jose, CA, USA) in Dulbecco's modified Eagle medium (DMEM; Invitrogen, Grand Island, NY, USA) supplemented with 10% fetal bovine serum (FBS; Hyclone, Thermo Scientific, Waltham, MA, USA), non-essential amino acids (MEM NEAA; Invitrogen) and penicillin-streptomycin-glutamine (PSG; Invitrogen) at 37°C in a humid-ified, 5% CO_2_ atmosphere. In some experiments, the HuES9.E1 cell line was transduced with green fluorescent protein (GFP) using lentivirus as the vector to obtain the GFP ES-MSC.

The NIH-3T3 and human bone marrow (BM) MSC (BM-MSC) cell lines were maintained on a tissue culture plate/flask surface (Becton Dickenson Falcon) in DMEM (Invitrogen) supplemented with 10% and 20% FBS (Hyclone, Thermo Scientific), respectively. The BM-MSC was isolated from consented donor BM from Singapore General Hospital (SGH, Singapore) as approved by the hospital ethics committee. The BM samples were plated at a density of 3×10^5^ cells/cm^2^ on tissue culture flask surfaces (Becton Dickenson Falcon) and grown till a confluent feeder layer was achieved. The BM-MSC cells were checked for expression of MSC markers such as CD44, CD73, CD90, CD105, CD166 and HLA-ABC (20,21).

Fresh UCB was obtained from Singapore Cord Blood Bank (SCBB), and the use of the UCB samples was reviewed and approved by the institutional review boards (IRB) of each cord blood collection hospital as well as those of the Singapore General Hospital (SGH, Singapore) and National University of Singapore (NUS, Singapore). The UCB-MNC were isolated using Ficoll Histopaque-1077 (Sigma Aldrich, St Louis, MO, USA) density-gradient centrifugation and counted before cyropreservation in 90% v/v donor autoplasma with 10% v/v dimethyl sulfoxide (DMSO; Sigma Aldrich) for subsequent use. CD34^+^ selected cells were obtained using Magnetic Activated Cell Sorting (MACS) cell-separation columns (Milte-nyi Biotec GmbH, Bergisch Gladbach, Germany). The cryopreserved UCB-MNC were thawed using a thawing solution containing human albumin (HAS; 20% w/v; Health Sciences Authority, Singapore) and Onkovertin 40 (10% w/v; B. Braun, Melsungen, Germany). The cells were centrifuged at 400 *g* for 15 min at 10 ° C. The cells were then washed with Dulbecco's phosphate-buffered saline (DPBS; Hyclone, Thermo Scientific), followed by centrifugation at 300 *g* for another 15 min. The cells were finally resus-pended in StemSpan-SFEM (STEMCELL Technologies, Vancouver, Canada) supplemented with 100 ng/mL human stem cell factor (SCF; PeproTech, Rocky Hill, NJ, USA), 100 ng/mL human throm-bopoietin (TPO; PeproTech, Rocky Hill, NJ, USA), 50 ng/mL human Flt3-Ligand (Flt3; PeproTech) and 20 ng/mL human insulin-like growth factor binding protein 2 (IGFBP2; R&D Systems, Minneapolis, MN, USA). The initial seeding density for human UCB-MNC was set at 2.5×10^5^cells/mL.

Viable cell counts of the ES-MSC, BM-MSC and NIH-3T3 were performed using trypan blue (Invitrogen), while crystal violet (Invitrogen) was used to count the nucleated UCB-MNC. A standard hemo-cytometer and upright microscope (bright field with 10 × magnification) were used.

### Stromal layer (ES-MSC, BM-MSC and NIH-3T3) and UCB-MNC co-cultures

For co-cultures that involved direct contact between the stromal layer and UCB-MNC, the ES-MSC, BM-MSC and NIH-3T3 were seeded at a density of 3.5×10^4^cells/cm^2^ in 1 mL media on 24-well plates (BD Falcon, Franklin Lakes, NJ, USA) and grown for 1 day (confluency >80%). Prior gelatin coating of the culture plates was required for the ES-MSC. The media were aspirated, followed by rinses with DPBS (Hyclone, Thermo Scientific), and 2.5×10^5^cell/mL UCB-MNC in StemSpan-SFEM (STEMCELL Technologies) containing the basal cytokines (SCF, TPO, Flt3 and IGFBP2) was inoculated to start the co-culture. UCB-MNC cultures without the stromal layer served as a control. The cultures were maintained in a humidified, 5% CO_2_ incubator at 37°C for 1, 2, 3, 7 and 11 days. Cytokine replenishment using the StemSpan-SFEM media was usually done on day 7 of the co-culture.

**Figure 4:**
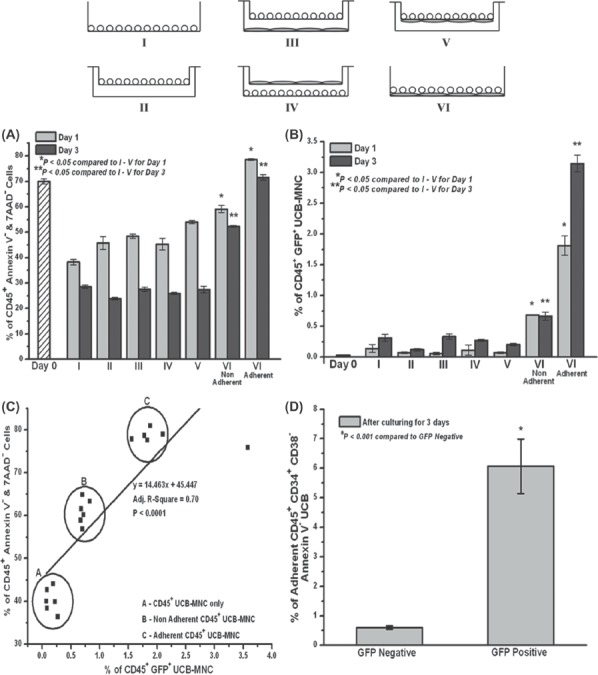
Contact dependency of the viability supporting effect and cytosolic transfer provided by the ES-MSC. (A) Viability supporting effect of the ES-MSC on UCB-MNC and (B) cytosolic transfer of the GFP from ES-MSC to UCB-MNC. Schematic diagram illustrating the locations of the two cell populations in the transwell system in the different experimental groups I–VI. (^*^P<0.05 compared with I–V for day 1 and ^*^P<0.05 compared with I–V for day 3). (C) Correlation between the amount of cytosolic transfer and UCB-MNC viability (^*^P<0.0001). (D) Phenotypic characterization of the adherent CD45^+^ GFP^+^ CD34^+^ CD38^-^ UCB-MNC in panel VI (^*^P<0.001). Data represent mean±from three independent experiments.

For co-cultures where ES-MSC were not in contact with UCB-MNC, a transwell insert (polyethylene terephthalate track-etched membrane, pore size 0.4 microns; BD Falcon) was used to separate these two cell populations in companion six-well plates (BD Falcon). ES-MSC-seeding parameters were the same as those described above except for group V (Figure 4), where the ES-MSC were grown on the lower side of the transwell membrane. In this case the seeding density of ES-MSC was increased three times. The transwells for group V (Figure 4) were incubated in the inverted position in a humidified, 5% CO_2_ incubator at 37°C for 2 h before placing them in the tissue culture plate for further incubation. ES-MSC culture media were aspirated, and the ES-MSC were rinsed with DPBS (Hyclone, Thermo Scientific) before adding 3.0 mL/well UCB-MNC containing StemSpan-SFEM media (STEMCELL Technologies). Transwell inserts were subsequently placed into each well at a height of 0.9 mm above the bottom of the well. UCB-MNC in transwell inserts without ES-MSC in the wells served as controls. Cultures were maintained in a humidified, 5% CO_2_ incubator at 37°C. On specified days, the cultures were harvested by washing with DPBS (Hyclone, Thermo Scientific).

**Figure 5:**
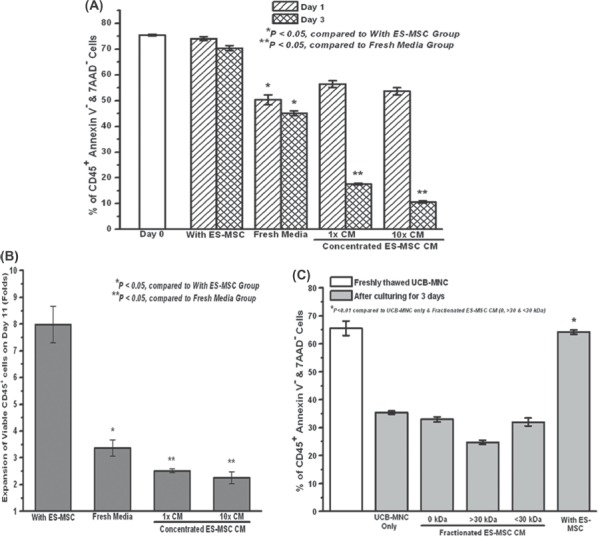
Absence of paracrine effect of culturing UCB-MNC with ES-MSC using CM (concentrated and fractionated) from ES-MSC. (A) Viability of UCB-MNC over days 1 and 3 when the UCB-MNC were cultured in the ES-MSC concentrated (using dialysis) CM and (B) expansion of UCB-MNC on day 11 when the UCB-MNC were cultured in the concentrated ES-MSC CM (^*^P<0.05 compared with the group with ES-MSC; ^**^P<0.05 compared with the group with fresh media). (C) UCB-MNC cultured in fresh ES-MSC CM that was fractionated using 30-kDa cut-off spin filters. The viability of the non-fractionated and fractionated portions (>30 kDa or <30 kDa) was measured at the end of a 3-day culture period compared with the direct ES-MSC co-culture system (^*^P0.01). Data represent the mean±SEM from three independent experiments.

For experiments requiring concentrated ES-MSC-conditioned media (CM) (Figure 5A, B), the culture media from the ES-MSC flask were collected after 3 days and concentrated using dialysis. For the fresh fractionated ES-MSC CM (Figure 5C), a Vivaspin 6 polyethersulfone column (Sartorius Stedim Biotech, France) with a molecular weight cutoff (MWCO) of 30 kDa was used. The two portions (>30 kDa and <30 kDa) of the fractionated CM were obtained after centrifugation of the VivaSpin 6 column at 4000 *g* in a swing bucket centrifuge.

### Separation of the combined, non-adherent and adherent fraction of the UCB-MNC after co-culture with the stromal layer

Upon co-culturing, the UCB-MNC that remained tightly attached to the ES-MSC (or BM-MSC and NIH-3T3) layer were defined as the adherent fraction of the UCB-MNC, while the rest, which were floating freely in the growth media, were defined as the non-adherent fraction of the UCB-MNC (22). In order to separate the two UCB-MNC fractions, the media were first gently removed from the wells, followed by a wash using DPBS (Hyclone, Thermo Scientific) to allow the collection of the non-adherent UCB-MNC from the co-culture system. For the adherent fraction, 0.5 mL Accutase (PAA Laboratories GmbH, Pasching, Austria) was added to the wells and incubated for 5 min at 37°C in a humidified 5% CO_2_ atmosphere. The wells were then washed with DPBS (Hyclone, Thermo Scientific) and the adherent fraction of the UCB-MNC, along with the ES-MSC, was collected. A combination of the non-adherent and adherent fraction was defined as the combined fraction. Harvesting of the combined fraction was similar to the non-adherent fraction except that more rigorous washing was carried out with the DPBS.

### Determination of intercellular transfer of cytosolic materials

The lentivirus GFP-expressing or quantum dot (QD) (Qtracker 525; Molecular Probes, Invitrogen, Grand Island, NY, USA)-labeled ES-MSC were seeded at a density of 7.5×10^4^ cells/well in 1 mL media in gelatin-coated 24-well plates (BD Falcon) and grown for 1 day. The media were aspirated, followed by rinsing with DPBS (Hyclone, Thermo Scientific, UT, USA), and 2.5×10^5^cell/mL UCB-MNC were seeded in 1 mL UCB expansion media. Cell cultures without the GFP ES-MSC layer and the GFP ES-MSC layer without the UCB-MNC served as controls. Cultures were maintained for 1, 2 and 3 days in the incubator (37°C in a humidified, 5% CO_2_ atmosphere) and the adherent and non-adherent UCB-MNC were harvested as described above. The labeling of the ES-MSC with QD (Molecular Probes, Invitrogen) was performed according to the manufacturer ’ s protocol.

For confocal imaging, GFP ES-MSC were co-cultured with CD34-selected UCB-MNC in ibidi μ dishes (Applied BioPhysics, Troy, NY, USA). CD34-selected UCB-MNC cultures without the GFP ES-MSC layer and the GFP ES-MSC layer without the CD34-selected UCB-MNC served as controls. Cultures were maintained in a humidified 5% CO_2_ incubator at 37°C for 4 days. A similar methodology was used to obtain the adherent and non-adherent fractions of the CD34-selected UCB-MNC. The collected CD34 cells were labeled with primary antibody CD45 (mouse-anti-human; BD Pharmingen, Franklin Lakes, CA, USA) (because an initial flow cytometer-based analysis of the CD34 cells showed 100% phenotypic expression of CD45) followed by a secondary antibody Alexa Fluor 568 (goat-anti-mouse; Molecular Probes, Invitrogen). The labeled CD34 cells were transferred to a microscopic slide by cytospin centrifugation and mounted using cover slips. Imaging (Z series) of the slides was done using a Carl Zeiss LSM710 system (40×oil immersion lens). The captured images were analyzed with ZEN 2009 Analysis software.

### Flow cytometry analyses

All data were acquired using a Cytomics FC500 Flow Cytometer (Beckman Coulter Inc., Brea, CA, USA) and 10 000 events per sample were collected. Acquired data were subsequently analyzed with CXP analysis software (Beckman Coulter Inc.). Isotype controls were used for the purposes of gating out non-specific antibody binding during analysis. Phycoerythrin (PE)-conjugated CD34 (CD34–PE; BD Pharmingen), allophycocyanin (APC)-conjugated CD38 (CD38–APC; BD Pharmingen) and fluo-rescein isothiocyanate-conjugated CD45 (CD45–FITC; BD Pharmingen) were used for CD45^+^ CD34^+^ CD38^−^ cell phenotype analysis, and FITC-conjugated Annexin-V (Annexin-V–FITC; Beckman Coulter Inc.), 7-amino-actinomycin D (7-AAD; Beckman Coulter Inc.) and PE–Cy7 conjugated CD45 (CD45–PE–Cy7; BD Pharmingen) were used for cell viability analysis. For the monitoring of mitochondrial membrane potential, harvested samples were stained with 1 mg/mL 5,5′,6,6′-tetrachloro-1,1′,3,3′ tetra-ethyl-benzimidazolyl-carbocyanine iodide (JC-1; Sigma Aldrich) at 37°C for 15–30 min, and were subsequently rinsed once with 2 mL warmed phosphate-buffered saline (PBS; Invitrogen) before data acquisition. For detecting the cytosolic transfer of GFP from the MSC to the UCB-MNC, the harvested UCB-MNC were stained with CD45–PE–Cy7 (BD Pharmingen). For the viability of the CD45^+^ GFP^+^ UCB cells, PE-conjugated Annexin V (Annexin-V–PE; BD Pharmingen) and APC-conjugated CD45 (CD45–APC; BD Pharmingen) were used. Further phenotypic characterization of the viable CD45^+^ GFP^+^ cells was carried out using Annexin-V–PE (BD Pharmingen), PE–Texas Red-conjugated CD34 (CD34–ECD; Beckman Coulter Inc.), CD38–APC and CD45–PE–Cy7 (BD Pharmingen).

To study the caspase activation in the UCB-MNC, Caspa-Tag Caspase 3/7, 8 and 9 (Millipore, Chemicon, Billerica, MA, USA) were used as per the manufacturer's protocol and the stained cells were analyzed using an FC500. For the cell-cycle analysis of the CD34-selected cells, a BD Cycletest Plus DNA reagent kit (BD Biosciences) was used as per the manufacturer's protocol. For analysis of the BM-MSC markers, the antibodies used were PE–Cy7-conjugated CD44 (CD44–PE–Cy7; BD Pharmingen, CA, USA), PE-conjugated CD73 (CD73–PE) and CD166 (CD166–PE; BD Pharmingen, CA, USA), FITC-conjugated CD90 (CD90–FITC) and CD105 (CD105–FITC) and Alexa Fluor 488-conjugated HLA-ABC (HLA-ABC-AF48; BD Pharmingen).

### Colony-forming unit assays

Granulocyte–macrophage colony-forming units (CFU-GM) from freshly thawed UCB-MNC or cells expanded for 11 days in the above culture systems were evaluated. Five thousand and 10 000 cells from freshly thawed UCB-MNC, and 1000 cells and 5000 cells from expanded cells, were cultured in duplicate in 35-mm tissue culture dishes (BD Falcon) in 1.1 mL 1% methylcellulose medium (Miltenyi Biotec GmbH) containing 30% FBS, 1% bovine serum albumin (BSA), 2 mM L-glutamine, 0.1 mM-mercaptoethanol, 50 ng/mL SCF, 20 ng/mL granulocyte–colony-stimulating factor (G-CSF), 20 ng/mL interleukin (IL)-3 and 20 ng/mL IL-6. After 14–16 days of culture in a humidified environment at 37°C in 5% CO_2_, colonies were scored using a SZ61 Olympus microscope (Olympus Europa GmbH, Planegg, Germany).

### Sorting of CD45^+^Annexin V^+^ 7-AAD^-^ and CD45^+^GFP^+^ UCB-MNC

The cryopreserved UCB-MNC were thawed by the process described above. The cells were then labeled with Annexin-V–FITC (Beckman Coulter Inc.), 7-AAD (Beckman Coulter Inc.) and CD45–PE–Cy7 (BD Pharmingen). To sort for the CD45^+^ GFP^+^ co-cultured UCB-MNC, the adherent and non-adherent UCB-MNC were obtained as described above. The cells were then labeled with CD45–PE–Cy7 (BD Pharmingen). The labeled cells were sorted using a BD FACsAriaII (Customized Unit at National Cancer Center, Singapore). Single-stained cells and fluorescent minus one (FMO) controls were used to determine the appropriate gating of the sort. The height and width of the forward and side scatters were used to eliminate tetraploid cells (see the supplementary Figure 1 to be found online at http://www.informahealthcare.com/doi/abs/10.3109/14653249.2012.697146). A 100-micron nozzle was used for the sort and the sorting efficiency was >90%.

**Figure 1:**
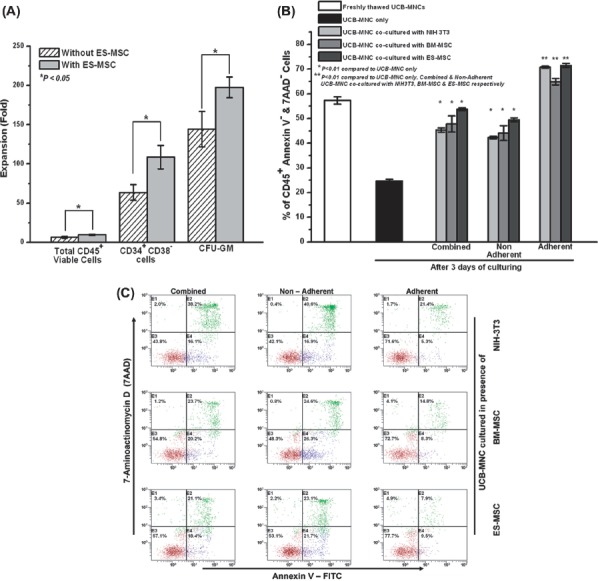

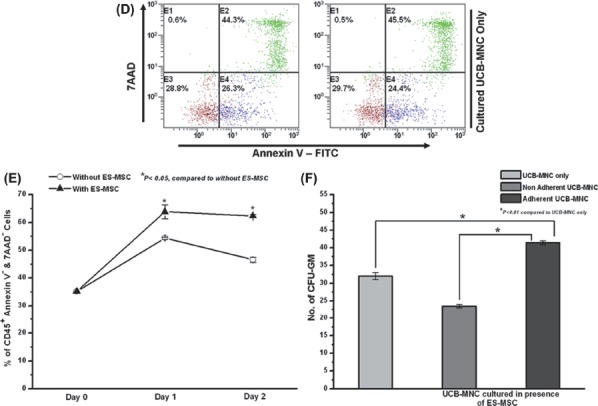
ES-MSC, BM-MSC and NIH-3T3 supported the viability of the Ficoll-separated UCB-MNC in a co-culture system. (A) Fold-expansion of total CD45^+^ viable cells, CD34^+^ CD38^-^ cells and CFU over a time–course of 11 days when UCB-MNC were co-cultured in the presence of ES-MSC. (B) Effect of stromal layer (NIH-3T3, BM-MSC and ES-MSC) co-culture on the percentage of viable CD45^+^ UCB-MNC (combined, non-adherent and adherent fractions) over a 3-day culture period (^*^P<0.01). (C) Representative flow cytometer plots showing the enhanced viability of the different fractions of UCB-MNC (combined, non-adherent and adherent) when co-cultured in the presence of the stromal layers NIH-3T3, BM-MSC and ES-MSC over a 3-day culture period. Viable CD45^+^ cells were those that were double-negative for Annexin V–FITC and 7-AAD (represented by brown dots in quadrant E3). Early apoptotic CD45^+^ cells were Annexin V–FITC-positive but 7-AAD-negative (represented by blue dots in quadrant E2). (D) Representative flow cytometer plots showing the reduced viability of the UCB-MNC when cultured without the stromal layer support over a 3-day culture period. (E) Effect of ES-MSC co-culture on the viability of flow-sorted early apoptotic CD45^+^ Annexin V^+^ UCB-MNC (combined fraction) over a 2-day culture period. Viable CD45^+^ cells were defined by double negativity for Annexin V–FITC and 7-AAD (^*^P<0.05). (F) CFU-GM assay of the adherent and non-adherent fraction of the UCB-MNC that was co-cultured with ES-MSC (^*^P<0.01). Data represent mean ± SEM from three independent experiments.

### DNA extraction and analysis of CD45^+^ GFP^+^ UCB-MNC

DNA of the CD45^+^ GFP^+^ UCB-MNC was determined by variable number tandem repeat (VNTR). Genomic DNA from the sorted CD45^+^ GFP^+^ UCB cells was extracted using a DNeasy blood and tissue kit (Qiagen, Valencia, CA, USA). Polymerase chain reaction (PCR) amplification of human-specific locus D1S80 (23) was performed using the following primers: 5′-GAAACTGGCCTCCAAACACTGCCCGCCG-3′ and 5′-CTTGTTGGAGATGCACGTGCCCCTTGC-3′.

PCR was carried out using the DyNAzyme™ EXT DNA polymerase (Finnzymes, Thermo Fisher Scientific, Vantaa, Finland) with the following conditions. DNA was first denatured at 95°C for 2 min, then it was amplified for five cycles at 95°C for 30 s, 62°C annealing for 30 s, and this was followed by one cycle of elongation at 72°C for 45 s and a final extension at 72°C for 10 min. Genomic DNA samples from non-co-cultured UCB-MNC and MSC were processed in parallel. Amplified PCR products were electrophoresed in 2.6% agarose (Invitrogen) gels at 80 V, stained with ethidium bromide (Sigma Aldrich), and visualized under ultraviolet (UV) light (Bio-Rad, Hercules, CA, USA).

### Data analyses

All the experiments were repeated at least three times, and data are presented as mean ± SEM. Statistical analyses included *t*-tests, with a *P*-value of <0.05 considered to be statistically significant.

## Results

### ES-MSC support ex vivo expansion and viability of UCB-MNC in co-culture

Previous studies have demonstrated that BM-MSC co-culture enhances *ex vivo* expansion of UCB-MNC (17,18,24,25). To confirm this observation under our experimental conditions, we first elucidated the effect of ES-MSC co-culture on *ex vivo* expansion of UCB-MNC. As shown in Figure 1A, the presence of the ES-MSC layer significantly increased the UCB-MNC expansion in terms of total viable cells (12.5 ±0.5 versus 8.2 ± 0.3), CD34^+^ CD38^-^ cells (107 ± 9 versus 61 ± 4) and CFU-GM populations (201 ± 14 versus 150 ± 20) over the time–course of 11 days.

UCB hematopoietic progenitor cells, which have been shown to have a higher self-renewal capacity as assessed by cell-surface markers and gene expression profile coupled with *in vivo* functional assays, have a greater tendency to adhere tightly to MSC in *in vitro* co-culture systems (22,26–28). As such, in our MSC-UCB co-culture system, we studied the behavior of the adherent, non-adherent and combined UCB fractions separately.

We investigated the viability of the CD45^+^ cells using the Annexin-V/7-AAD double-staining method by flow cytometer, where viable cells are defined as double negative for Annexin-V and 7-AAD. We demonstrated that immediately after thawing the UCB-MNC, the viability was 57.3 ± 1.5%. After a 3-day culture period without co-culture, the UCB-MNC viability decreased to 24.6 ± 0.7% (Figure 1B, D). In contrast, when UCB-MNC were co-cultured with ES-MSC, BM-MSC or NIH-3T3 (i.e. with stromal support), the percentage of viable CD45 ^+^ cells was significantly higher in the combined, non-adherent and adherent fractions of the UCB-MNC (Figure 1B, C). In the presence of ES-MSC, the viability of the combined, non-adherent and adherent UCB-MNC was 53.7 ± 0.6%, 49.5 ± 0.8% and 71.5 ± 0.9%, respectively (Figure 1B). The NIH-3T3 and BM-MSC co-culture system also demonstrated a similar viability, enhancing the effect on the co-cultured UCB-MNC (Figure 1B, C). In all our experiments, the adherent CD45^+^ cells exhibited the highest viability compared with the other fractions of co-cultured UCB-MNC. Interestingly, the adherent cells from the ES-MSC, BM-MSC and NIH-3T3 co-culture had a reduction in early apoptotic cells (CD45^+^ Annexin-V^+^) to less than 10% (Figure 1C).

To confirm further the viability of the supporting effect of the ES-MSC, we sorted for the early apoptotic CD45^+^ Annexin-V^+^ UBC-MNC from a freshly UCB thawed sample (Figure 1E) and co-cultured the sorted cells with ES-MSC. As shown in Figure 1E, the co-cultured group (62.4 ± 0.5%) had a significantly higher population of viable CD45^+^ Annexin V^-^ cells compared with the non-co-cultured group (46.6 ± 0.9%) on day 2.

We further investigated the colony-forming ability of the non-adherent and adherent UCB-MNC (27). The adherent UCB-MNC formed significantly higher numbers of CFU-GM (41.5 ± 0.5) than the non-adherent fraction (23.5 ±0.5) (Figure 1F).

### The presence of ES-MSC prevented the loss of mitochondrial membrane potential in UCB-MNC

**Figure 2:**
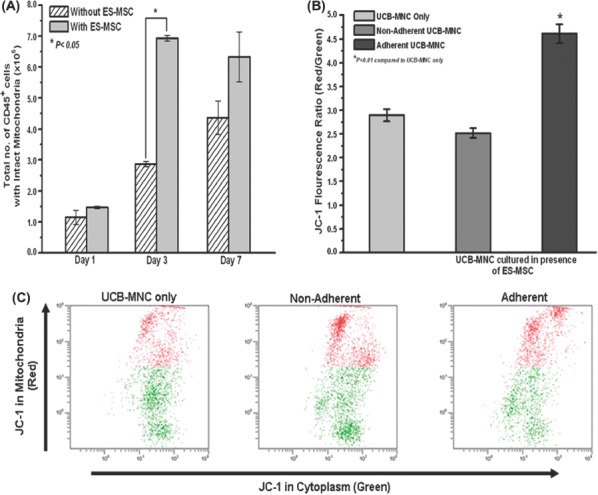
Effect of ES-MSC co-culture on the mitochondrial membrane potential of the UCB-MNC. (A) The mitochondrial membrane potential of UCB-MNC (combined fraction) over a time-course of 7 days, as determined by JC-1 staining. The number of cells with intact mitochondria was calculated from the red fluorescence-positive portion multiplied by the total number of cells (^*^P<0.05). (B) JC-1 red to green fluorescence ratio (red fluorescence mean/green fluorescence mean) in the non-adherent and adherent fractions of the UCB-MNC co-cultured with ES-MSC and non-co-cultured UCB-MNC over a 3-day culture period (^*^P<0.01). (C) Representative flow cytometer plots of the JC-1 staining performed on the non-adherent and adherent fractions of co-cultured (with ES-MSC) UCB-MNC compared with the non-co-cultured control. Data represent mean±SEM from three independent experiments.

To determine whether the observed viability support effect of ES-MSC on UCB-MNC was correlated with the extent of loss of mitochondrial membrane potential, the mitochondrial membrane potential was studied using a JC-1 assay (Figure 2A). In healthy cells, JC-1, being a positively charged lipophilic dye, enters the intact and negatively charged mitochondrial membrane matrix, where it accumulates to form the red fluorescent J-aggregate. However, in apoptotic cells the mitochondrial membrane potential collapses, thus preventing the accumulation of JC-1 in the mitochondrion and formation of the J-aggregate. Thus the green fluorescent monomeric form remains in the cytoplasm of the apoptotic cells. The ratio of the red to green fluorescence of a JC-1-stained cell, as measured with a flow cytometer, can be used to assess the mitochondrial-dependent apoptotic state of cells. In contrast with UCB-MNC cultured in the absence of ES-MSC (3.0×1^5^±0.2), a 3-day co-culture with ES-MSC (6.8×10^5^±0.2) significantly increased the number of UCB-MNC that exhibited intact mitochondrial membrane potential. In addition, the adherent UCB-MNC (4.6±0.2) had a significantly higher JC-1 fluorescence ratio (red/green) compared with the non-adherent cells (2.5±0.2) and the UCB-MNC without co-culture (Figure 2B, C). We also observed a significant reduction in caspase 3/7 and 9 activity when the UCB-MNC were co-cultured with ES-MSC (see the supplementary Figure 2A to be found online at http://www.informahealthcare.com/doi/abs/10.3109/14653249.2012.697146). From the cell-cycle analyses of the CD34-selected cell population, the presence of the ES-MSC layer resulted in a significant increase in the percentage of cells in the S and G2/M phase on day 1 (see the supplementary Figure 2B, C to be found online at http://www.informahealth-care.com/doi/abs/10.3109/14653249.2012.697146). Taken together, these results supported the ability of ES-MSC to rescue the thawed UCB-MNC from early apoptosis by preventing the loss of mitochondrial membrane potential.

### Transfer of cytosolic components from the ES-MSC to the UCB-MNC

**Figure 3:**
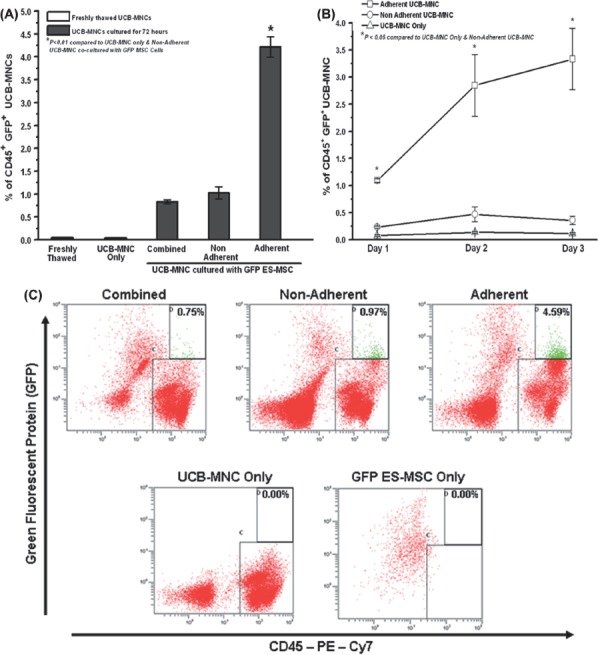

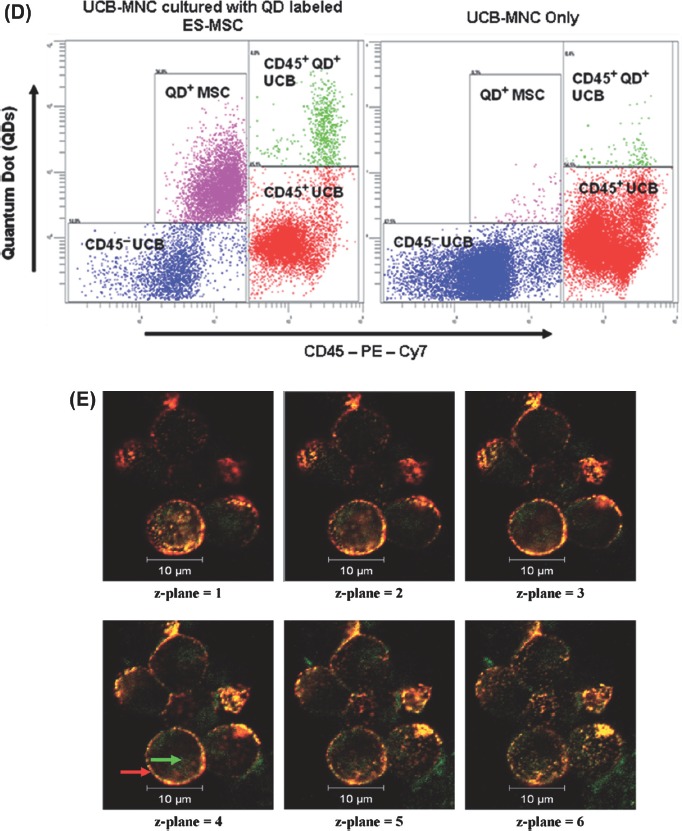
Transfer of cytosolic GFP from GFP-expressing ES-MSC to adherent UCB-MNC under direct-contact co-culture. (A) Percentage of CD45^+^ GFP^+^ cells in the combined, non-adherent and adherent fractions of the co-cultured (with GFP ES-MSC) UCB-MNC and non-co-cultured UCB-MNC over a 3-day culture period (^*^P<0.01). (B) Time–course study of the cytosolic transfer of GFP from the ES-MSC to the non-adherent and adherent UCB-MNC (^*^P<0.01). (C) Representative flow cytometer plots of the CD45–PE–Cy7-labeled combined, non-adherent and adherent UCB-MNC co-cultured with GFP ES-MSC. The CD45^+^ GFP^+^ population is depicted by the green dots (quadrant D). Representative plots for non-co-cultured UCB-MNC and only GFP ES-MSC stained with CD45–PE–Cy7 are also shown. (D) Representative plot for adherent UCB-MNC co-cultured with QD-labeled ES-MSC. The CD45^+^ QD^+^ population is represented by the green dots in the appropriately labeled quadrant. (E) Representative Z-series confocal images of three independent experiments demonstrating the transfer of GFP from the GFP-labeled ES-MSC to the UCB-MNC. The adherent UCB-MNC were co-cultured with GFP ES-MSC for 4 days. The UCB-MNC were first stained with the primary antibody for CD45 followed by the secondary antibody Alexa Fluor 568. Z-planes 1–6 represent consecutive Z-planes taken during fluorescence confocal imaging, and Z-planes 1 and 6 represent the outer surface of the cell membrane (imaged using Carl Zeiss LSM710 with a 40×oil immersion lens; Z-plane thickness=0.44 μm). Data represent mean±SEM from three independent experiments.

Lentivirus-transduced ES-MSC expressing intracellular GFP were co-cultured in direct contact with UCB-MNC to elucidate the possibility of transfer of cytosolic components from the ES-MSC to the UCB-MNC. As depicted in Figure 3A, C, the adherent UCB-MNC (4.2±0.2%) had a significant transfer of GFP from the ES-MSC, as evidenced by the presence of the CD45^+^ GFP^+^ population by flow cytometry analysis. There was no background GFP fluorescence observed in the control groups, which included non-co-cultured UCB-MNC and ES-MSC alone; in addition, significantly lower percentages of transfer-positive cells were observed in the combined (0.8 ±0.04%) and non-adherent (1.0 ±0.1%) fractions over a 3-day culture period (Figure 3A, C).

A more detailed study of the adherent population revealed that the percentage of CD45^+^ GFP^+^ cells increased from 1.0±0.03% to 3.3±0.6% from day 1 to day 3 (Figure 3B). This provided strong evidence of the transfer of cytosolic material from ES-MSC to UCB-MNC over the 3-day co-culture period, in which the UCB-MNC CD45^+^ cells became positive for green fluorescence, which was not initially present in them. For further confirmation of cytosolic transfer, ES-MSC were labeled with QD and co-cultured with CD34-selected UCB-MNC. Within 24 h of co-culture, the UCB-MNC (CD45^+^ cells) became positive for QD (Figure 3D).

Moreover, to ensure that the GFP marker was actually enclosed by the CD45^+^ cells, we carried out

Z-series imaging of the CD45^+^ GFP^+^ cells using fluorescent confocal microscopy (Figure 3E). The outer surface of the cell membrane was predominantly red because of the labeling of the CD45 antigen by CD45 (primary) and Alexa Fluor 568 (secondary) antibody. As we probed inside the cell (Z-plane 4; Figure 3E) we were able to detect the presence of the intracellular GFP (green arrow) bound by the red cell membrane (red arrow) of the CD45 cells.

### The viability supporting effect of ES-MSC and cytosolic transfer from the ES-MSC to early HPC in UCB-MNC was most prominent when ES-MSC and UCB-MNC were in direct contact

To investigate whether direct contact between UCB-MNC and ES-MSC was necessary to prevent the UCB-MNC from undergoing early apoptosis and maintaining their viability, we cultured the UCB-MNC under three different conditions: in the presence of ES-MSC that were in direct contact with UCB-MNC (Figure 4; group VI), in the presence of ES-MSC but with the two cell populations separated by a transwell insert (Figure 4; groups III and V), and in the absence of ES-MSC co-culture (Figure 4; groups I and II). As demonstrated in Figure 4A, the optimal viability support for both the non-adherent (52.2±0.3%) and adherent (71.5±1.1%) fractions was achieved when the UCB-MNC were in direct contact with the ES-MSC layer on day 3. Large (0.9 mm) (groups III and IV) or small (membrane thickness of the transwell) (group V) physical separation of the two populations of cells by the transwell also significantly reduced the viability. Furthermore, the use of a non-viable MSC layer, whereby the MSC cells were fixed with paraformaldehyde or by freezing, did not enhance UCB viability and its subsequent expansion (data not shown). Similarly, cytosolic transfer of GFP from the ES-MSC to the adherent UCB-MNC occurred only when the two cell populations were in direct contact with each other (Figure 4B), with negligible transfer taking place in the other groups. A statistical correlation between higher levels of cytosolic transfer and enhanced UCB cell viability was observed (Figure 4C).

A detailed phenotypic characterization of the viable adherent CD45^+^ cells revealed that transferpositive (or GFP^+^) cells (6.1±0.9%) expressed a significantly higher percentage of the CD34^+^ CD38^-^ profile compared with the transfer-negative population (0.6±0.05%) (Figure 4D).

### Enhanced UCB-MNC viability is not the result of a paracrine effect or transdifferentiation of ES-MSC

Several research groups have reported that MSC helps in tissue regeneration and repair via the release of small biomolecules (29–34) (paracrine effect) or by transdifferentiating (35–38) into the required cell types. As such, we investigated whether these mechanisms could have played a role in the viability enhancement of the UCB-MNC. Concentrated CM from ES-MSC was used to culture the UCB-MNC cells (Figure 5A, B). As seen in Figure 5A, the UCB-MNC viability cultured in the ES-MSC-concentrated (1×) CM (17.6±0.3%) showed a significant reduction compared with the co-cultured group (70.4±1.0%) after a 3-day culture period. Furthermore, in order to study the effect of the CM concentration, a higher (10×) concentrated CM was used, and a similar decrease in viability (10.7±0.1%) was observed. The low viability of the UCB-MNC in the concentrated CM groups (1× and 10×) translated to a significantly lower expansion of the viable CD45^+^ cells over an 11-day culture (Figure 5B).

A similar experiment was repeated using fresh ES-MSC CM that was fractionated using a 30-kDa MWCO membrane. The viability of the UCB-MNC cultured in the >30-kDa fraction (24.7±0.8%) and <30-kDa fraction (32.0±1.4%) was significantly lower than the direct ES-MSC co-cultured control (64.2±0.8%) in a 3-day culture system.

**Figure 6:**
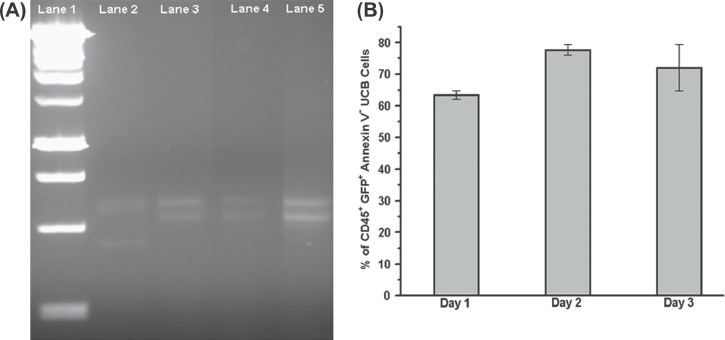
DNA and viability analysis of the cytosolic transfer-positive UCB-MNC. (A) To exclude MSC transdifferentiation and MSC-UCB fusion as the reasons for the presence of CD45^+^ GFP^+^ cells, VNTR using PCR amplification at human locus D1S80 was carried out on the extracted DNA of the sorted CD45^+^ GFP^+^ cells. Lane 1, 1 kbp DNA ladder. Lane 2, GFP expressing ES-MSC. Lane 3, non-co-cultured UCB-MNC. Lane 4, sorted CD45^+^ GFP^+^ cells obtained after co-culturing for 3 days with GFP ES-MSC. Lane 5, sorted CD45^+^ cells obtained after co-culturing for 3 days with GFP ES-MSC. As shown, the band for CD45^+^ GFP^+^ cells (lane 4) corresponds with that of UCB-MNC only (lane 3). (B) Percentage of adherent CD45^+^ GFP^+^ UCB-MNC that are Annexin V^-^ when co-cultured with GFP ES-MSC over a 3–day time–course. Data represent mean±SEM from three independent experiments.

Finally, to rule out the possibility of cell fusion between the GFP-expressing ES-MSC and CD45^+^ UCB-MNC or transdifferentiation of the ES-MSC to give rise to a CD45^+^ GFP^+^ population, we performed fluorescence-activated cell sorting to purify the specific diploid CD45^+^ GFP^+^ population from the MSC-UCB co-culture system. Analysis of the extracted DNA was performed using VNTR at the polymorphic human locus D1S80. VNTR, which are repeated DNA sequences, are arranged in tandem with 7 - 100 base pairs. Thus the number of repeats at the VNTR locus is unique for the MSC and UCB cells used in the co-culture system, thereby enabling the recognition of the genetic identity of the respective cell population. PCR amplification of D1S80 and agarose gel electrophoresis revealed that the amplified DNA of the CD45^+^ GFP^+^ cells corresponded with the DNA pattern of the UCB-MNC (Figure 6A), thus suggesting that the transfer GFP^+^ cells was of UCB-MNC origin but not an outcome of MSC transdifferentiation or MSC-UCB cell fusion. Also, a detailed assessment of the viability of CD45^+^ GFP^+^ cells revealed that approximately 70–80% of the transfer of GFP^+^ cells were viable over a time–course of 3 days (Figure 6B). The CD45^+^ GFP^+^ were also able to form colony forming units (supplementary Figure 3 to be found online at http://www.informahealthcare.com/doi/abs/10.3109/14653249.2012.697146).

## Discussion

To our knowledge, this is the first study that addresses intercellular transfer as a mechanism of MSC enhancing the viability of UCB cells during *ex vivo* expansion. We have demonstrated that MSC co-culture supports the viability of frozen-thawed UCB cells, and this effect was best seen in the presence of cell – cell contact with MSC. We have also shown that the transfer of cytosolic materials occurs between MSC and co-cultured UCB cells, and that a statistically significant positive correlation exists between cytosolic transfer and enhanced UCB cell viability.

Our data indicate that the viability supporting effect of stromal cells (ES-MSC, BM-MSC as well as NIH-3T3) can be mediated through the reversal of early apoptotic events in UCB-MNC, as demonstrated by the reduction in phosphatidylserine externalization coupled with the prevention of the loss of mitochondrial membrane potential and reduction of caspase activation. The viability supporting effect of the ES-MSC was optimal only during direct contact, with adherent UCB-MNC exhibiting the highest viability, illustrating that secreted factors are not likely to be the most important determinant, but rather a cell-to-cell contact-dependent process might be involved. The use of concentrated or fractionated ES-MSC CM did not exhibit a similar viability supporting effect on the UCB-MNC. The use of a GFP-labeled ES-MSC layer indicated that intercellular transfer of cytosolic components from MSC to UCB-MNC is possible, and that the extent of transfer of GFP from MSC to UCB-MNC appears to be dependent on the contact of these two cell populations, which also correlates with the viability supporting effect. We were also able to identify the intercellular transfer taking place in primitive, viable HPC populations, as defined by phenotypic markers such as CD34 and CD38. In another study, Spees *et al.* (39) reported that cells could rescue surrounding cells from apoptosis by direct intercellular transfer of mitochondria. The phenomenon of direct mitochondria transfer between cells could play a role in the stromal cell viability supporting effect that has been observed in MSC–cardiomyocyte co-culture systems, as ischemic cardiomyocytes were rescued from cell death (40–43), which is an observation similar to our current UCB *ex vivo* expansion system.

Similar observations of cytosolic transfer have been reported by Gillette *et al.* (44), who identified that the transfer of cytosolic materials from HPC to osteoblasts plays an important role in the production of stromal-derived factor-1 (SDF-1) by the osteoblasts, which in turn control HPC homing in the BM. Therefore it may be reasonable to suggest that the process of cytosolic transfer is bi-directional, with multiple biologic functions that help in regulating the *ex vivo* osteoblastic and vascular niche (45).

Recently, intercellular communications mediated by tunneling nanotubes (46,47) and exosomes (48,49) have gained significant importance in explaining crucial physiologic and biologic processes that include cell death (50), transfer of virus (51), organelles (51) and mRNAs (52). Valadi *et al.* (52) have demonstrated the transfer of functional genetic materials in the form of mRNAs and microRNAs through exosomes.

According to our data, direct contact accompanied by the adhesion of UCB-MNC to the MSC layer is the single most important factor in regulating the intercellular transfer of GFP from the ES-MSC to the UCB-MNC. This observation is in accordance with those made by Gillette *et al.* (44), who reported that cytosolic transfer from HPC to the osteoblasts would only occur when the two cell layers were in direct contact with each other. Moreover, Wagner *et al.* (22,27) have demonstrated that HPC that have a significantly higher self-renewing ability (CD34^+^ CD38^-^ as opposed to CD34^+^ CD38^+^) dominate the adherent fraction. In these studies, uropod formation by the HPC (which was also observed in our co-culture system) to anchor itself to the MSC was described as the main mechanism of cellular contact and adhesion between the two cell layers (22,27,44,53,54). Adhesion proteins such as N-cadherin, cadherin-11 and very late antigen-4 (VLA-4) are also known to be highly expressed in the adherent portion compared with the non-adherent portion (44,45). Furthermore, *in vivo* studies involving connexin 43 (C×43)-deficient mice have shown that gap junctions are critical in regulating hematopoiesis in BM and thymus, via gap junction-mediated intercellular communications (53). In addition, ECM (consisting mainly of collagen and fibrinogen) created by the viable MSC layer may help in maintaining the viability of the UCB-MNC by mimicking an *in vitro* hematopoietic stem cell niche (55).

Based on the current data, it would be important to identify the type of cellular elements, signaling molecules, soluble factors, nutrients and/or organelles, that are transferred between the ES-MSC layer and UCB-MNC, as the identification of the cellular elements would shed new light on how these two cell populations communicate and the mechanism possibly responsible for the viability supporting effect of ES-MSC. Based on the size of the surrogate markers GFP and QD, which were shown to transfer from the ES-MSC to the UCB-MNC, it may be reasonable to suggest that molecules such as nutrients, antioxi-dants or even mRNAs, which are much smaller than GFP or QD, could also transfer in great quantity and mediate the biologic response of higher viability in the UCB-MNC.

In conclusion, our current study has demonstrated the transfer of cytosolic content from ES-MSC to UCB-MNC, and this phenomenon might mediate the reversal of early apoptosis of UCB. Further investigation of the intercellular communication between ES-MSC and UCB cells would shed new light on the mechanism by which ES-MSC supports UCB-MNC expansion and would open up new directions in the study of surface molecules and subsequent signaling.

## References

[1] Leo AJ, Grande DA (2006). Mesenchymal stem cells in tissue engineering. Cells Tissues Organs.

[2] Granero-Molto F, Weis JA, Longobardi L, Spagnoli A (2008). Role of mesenchymal stem cells in regenerative medicine: application to bone and cartilage repair. Expert Opin Biol Ther.

[3] Chen X, Armstrong MA, Li G (2006). Mesenchymal stem cells in immunoregulation. Immunol Cell Biol.

[4] Riekstina U, Cakstina I, Parfejevs V, Hoogduijn M, Jankovskis G, Muiznieks I (2009). Embryonic stem cell marker expression pattern in human mesenchymal stem cells derived from bone marrow, adipose tissue, heart and dermis. Stem Cell Rev.

[5] Chen HH, Decot V, Ouyang JP, Stoltz JF, Bensoussan D, de Isla NG (2009). In vitro initial expansion of mesenchymal stem cells is influenced by the culture parameters used in the isolation process. Biomed Mater Eng.

[6] Pittenger MF, Mackay AM, Beck SC, Jaiswal RK, Douglas R, Mosca JD (1999). Multilineage potential of adult human mesenchymal stem cells. Science.

[7] Rallapalli S, Bishi DK, Verma RS, Cherian KM, Guhathakurta S (2009). A multiplex PCR technique to characterize human bone marrow derived mesenchymal stem cells. Biotechnol Lett.

[8] Le Blanc K, Rasmusson I, Sundberg B, Götherström C, Hassan M, Uzunel M (2004). Treatment of severe acute graft-versus-host disease with third party haploidentical mesenchymal stem cells. Lancet.

[9] Ball LM, Bernardo ME, Roelofs H, Lankester A, Cometa A, Egeler RM (2007). Cotransplantation of ex vivo expanded mesenchymal stem cells accelerates lymphocyte recovery and may reduce the risk of graft failure in haploidentical hematopoietic stem-cell transplantation. Blood.

[10] Ringden O, Keating A (2011). Mesenchymal stromal cells as treatment for chronic GVHD. Bone Marrow Transplant.

[11] Giordano A, Galderisi U, Marino IR (2007). From the laboratory bench to the patient's bedside: an update on clinical trials with mesenchymal stem cells. J Cell Physiol.

[12] Aggarwal S, Pittenger MF (2005). Human mesenchymal stem cells modulate allogeneic immune cell responses. Blood.

[13] Lepperdinger G, Brunauer R, Jamnig A, Laschober G, Kassem M (2008). Controversial issue: is it safe to employ mesenchymal stem cells in cell-based therapies?. Exp Gerontol.

[14] Li QM, Fu YM, Shan ZY, Shen JL, Zhang XM, Lei L (2009). MSCs guide neurite directional extension and promote oli-godendrogenesis in NSCs. Biochem Biophys Res Commun.

[15] Tabera S, Pérez-Simón JA, Díez-Campelo M, Sánchez-Abarca LI, Blanco B, López A (2008). The effect of mesenchymal stem cells on the viability, proliferation and differentiation of B-lymphocytes. Haematologica.

[16] Choo A, Ngo AS, Ding V, Oh S, Kiang LS (2008). Autogeneic feeders for the culture of undifferentiated human embryonic stem cells in feeder and feeder-free conditions. Methods Cell Biol.

[17] McNiece I, Harrington J, Turney J, Kellner J, Shpall EJ (2004). Ex vivo expansion of cord blood mononuclear cells on mesenchymal stem cells. Cytotherapy.

[18] Robinson SN, Ng J, Niu T, Yang H, McMannis JD, Karandish S (2006). Superior ex vivo cord blood expansion following co-culture with bone marrow-derived mesenchymal stem cells. Bone Marrow Transplant.

[19] Lian Q, Lye E, Suan Yeo K, Khia Way Tan E, Salto-Tellez M, Liu TM (2007). Derivation of clinically compliant MSCs from CD105^+^, CD24^−^ differentiated human ESCs. Stem Cells.

[20] Totey S, Totey S, Pal R, Pal R (2009). Adult stem cells: a clinical update. J Stem Cells.

[21] Ferrari M, Corradi A, Lazzaretti M, De'Cillà M, Losi CG, Villa R (2007). Adult stem cells: perspectives for therapeutic applications. Vet Res Commun.

[22] Wagner W, Wein F, Roderburg C, Saffrich R, Faber A, Krause U (2007). Adhesion of hematopoietic progenitor cells to human mesenchymal stem cells as a model for cell-cell interaction. Exp Hematol.

[23] Budowle B, Chakraborty R, Giusti AM, Eisenberg AJ, Allen RC (1991). Analysis of the VNTR locus D1S80 by the PCR followed by high-resolution PAGE. Am J Hum Genet.

[24] Delalat B, Pourfathollah AA, Soleimani M, Mozdarani H, Ghaemi SR, Movassaghpour AA (2009). Isolation and ex vivo expansion of human umbilical cord blood-derived CD34^+^ stem cells and their cotransplantation with or without mesenchymal stem cells. Hematology.

[25] Robinson SN, Simmons PJ, Yang H, Alousi AM, Marcos de Lima J, Shpall EJ (2011). Mesenchymal stem cells in ex vivo cord blood expansion. Best Pract Res Clin Haematol.

[26] Alakel N, Jing D, Muller K, Bornhauser M, Ehninger G, Ordemann R (2009). Direct contact with mesenchymal stromal cells affects migratory behavior and gene expression profile of CD133^+^ hematopoietic stem cells during ex vivo expansion. Exp Hematol.

[27] Wagner W, Saffrich R, Wirkner U, Eckstein V, Blake J, Ansorge A (2005). Hematopoietic progenitor cells and cellular microen-vironment: behavioral and molecular changes upon interaction. Stem Cells.

[28] Wein F, Pietsch L, Saffrich R, Wuchter P, Walenda T, Bork S (2010). N-cadherin is expressed on human hematopoietic progenitor cells and mediates interaction with human mesenchymal stromal cells. Stem Cell Res.

[29] Xiang MX, He AN, Wang JA, Gui C (2009). Protective paracrine effect of mesenchymal stem cells on cardiomyocytes. J Zhejiang Uni Sci B.

[30] Xu RX, Chen X, Chen JH, Han Y, Han BM (2009). Mesenchymal stem cells promote cardiomyocyte hypertrophy in vitro through hypoxia-induced paracrine mechanisms. Clin Exp Pharmacol Physiol.

[31] Timmers L, Lim SK, Arslan F, Armstrong JS, Hoefer IE, Doevendans PA (2007). Reduction of myocardial infarct size by human mesenchymal stem cell conditioned medium. Stem Cell Res.

[32] Lai RC, Arslan F, Tan SS, Tan B, Choo A, Lee MM (2010). Derivation and characterization of human fetal MSCs: an alternative cell source for large-scale production of cardioprotective microparticles. J Mol Cell Cardiol.

[33] Lai RC, Arslan F, Lee MM, Sze NSK, Choo A, Chen TS (2010). Exosome secreted by MSC reduces myocardial ischemia/reperfusion injury. Stem Cell Res.

[34] Gnecchi M, Zhang Z, Ni A, Dzau VJ (2008). Paracrine mechanisms in adult stem cell signaling and therapy. Circ Res.

[35] Moviglia GA, Varela G, Gaeta CA, Brizuela JA, Bastos F, Saslavsky J (2006). Autoreactive T cells induce in vitro BM mesenchymal stem cell transdifferentiation to neural stem cells. Cytotherapy.

[36] Sasaki M, Abe R, Fujita Y, Ando S, Inokuma D, Shimizu H (2008). Mesenchymal stem cells are recruited into wounded skin and contribute to wound repair by transdifferentiation into multiple skin cell type. J Immunol.

[37] Phinney DG, Prockop DJ (2007). Concise review. Mesenchymal stem/multipotent stromal cells: the state of transdifferentiation and modes of tissue repair. Current views. Stem Cells.

[38] Ishikawa F, Shimazu H, Shultz LD, Fukata M, Nakamura R, Lyons B (2006). Purified human hematopoietic stem cells contribute to the generation of cardiomyocytes through cell fusion. FASEB J.

[39] Spees JL, Olson SD, Whitney MJ, Prockop DJ (2006). Mitochondrial transfer between cells can rescue aerobic respiration. Proc Natl Acad Sci USA.

[40] Plotnikov EY, Khryapenkova TG, Vasileva AK, Marey MV, Galkina SI, Isaev NK (2008). Cell-to-cell cross-talk between mesenchymal stem cells and cardiomyocytes in co-culture. J Cell Mol Med.

[41] Acquistapace A, Bru T, Lesault PF, Figeac F, Coudert AE, le Coz O (2011). Human mesenchymal stem cells reprogram adult cardiomyocytes toward a progenitor-like state through partial cell fusion and mitochondria transfer. Stem Cells.

[42] Cselenyák A, Pankotai E, Horváth EM, Kiss L, Lacza Z (2010). Mesenchymal stem cells rescue cardiomyoblasts from cell death in an in vitro ischemia model via direct cell-to-cell connections. BMC Cell Biol.

[43] Rechavi O, Goldstein I, Kloog Y (2009). Intercellular exchange of proteins: the immune cell habit of sharing. FEBS Lett.

[44] Gillette JM, Larochelle A, Dunbar CE, Lippincott-Schwartz J (2009). Intercellular transfer to signalling endosomes regulates an ex vivo bone marrow niche. Nat Cell Biol.

[45] Wein F, Pietsch L, Saffrich R, Wuchter P, Walenda T, Bork S (2010). N-cadherin is expressed on human hematopoietic progenitor cells and mediates interaction with human mesenchymal stromal cells. Stem Cell Research.

[46] Bukoreshtliev NV, Wang X, Hodneland E, Gurke S, Barroso JFV, Gerdes HH (2009). Selective block of tunneling nanotube (TNT) formation inhibits intercellular organelle transfer between PC12 cells. FEBS Lett.

[47] Gerdes HH, Bukoreshtliev NV, Barroso JFV (2007). Tunneling nanotubes: a new route for the exchange of components between animal cells. FEBS Lett.

[48] Théry C, Zitvogel L, Amigorena S (2002). Exosomes: composition, biogenesis and function. Nat Rev Immunol.

[49] Février B, Raposo G (2004). Exosomes: endosomal-derived vesicles shipping extracellular messages. Curr Opin Cell Biol.

[50] Arkwright PD, Luchetti F, Tour J, Roberts C, Ayub R, Morales AP (2010). Fas stimulation of T lymphocytes promotes rapid intercellular exchange of death signals via membrane nano-tubes. Cell Res.

[51] Gurke S, Barroso JFV, Gerdes HH (2008). The art of cellular communication: tunneling nanotubes bridge the divide. Histochem Cell Biol.

[52] Valadi H, Ekström K, Bossios A, Sjöstrand M, Lee JJ, Lötvall JO (2007). Exosome-mediated transfer of mRNAs and microRNAs is a novel mechanism of genetic exchange between cells. Nat Cell Biol.

[53] Montecino-Rodriguez E, Dorshkind K (2001). Regulation of hematopoiesis by gap junction-mediated intercellular communication. J Leukoc Biol.

[54] Giebel B, Corbeil D, Beckmann J, Höhn J, Freund D, Giesen K (2004). Segregation of lipid raft markers including CD133 in polarized human hematopoietic stem and progenitor cells. Blood.

[55] Wagner W, Saffrich R, Ho AD (2008). The stromal activity of mesen-chymal stromal cells. Trans Med Hemother.

