# Supplementary material for: Intercellular cytosolic transfer correlates with mesenchymal stromal cell rescue of umbilical cord blood cell viability during ex vivo expansion
Source: Cytotherapy. 2012 Jul 10;14(9):1064–79. doi: 10.3109/14653249.2012.697146 (PMC3484967; doi:10.3109/14653249.2012.697146)
Supplement: Supplementary file 1 [file mcyt14-1064-SD1.pdf]

Supplementary material for Chu et al., Intercellular cytosolic transfer correlates with mesenchymal stromal cell rescue of umbilical cord blood cell viability during *ex vivo* expansion. Cytotherapy, 2012;14:1064–1079.

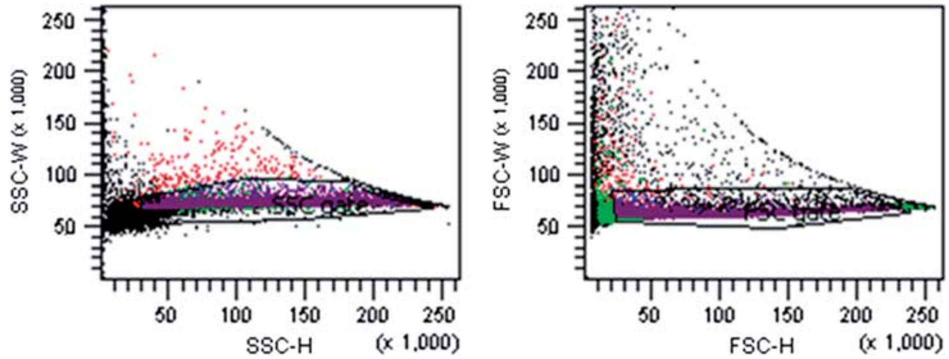

Supplementary Figure 1. The height and width of the forward (FSC) and side (SSC) scatter of the CD45^{+} GFP^{+} cells. The parameters are used to demonstrate no cell doublets arising from UCB – MSC fusion. Data represent mean ± S.E.M. from three independent experiments.

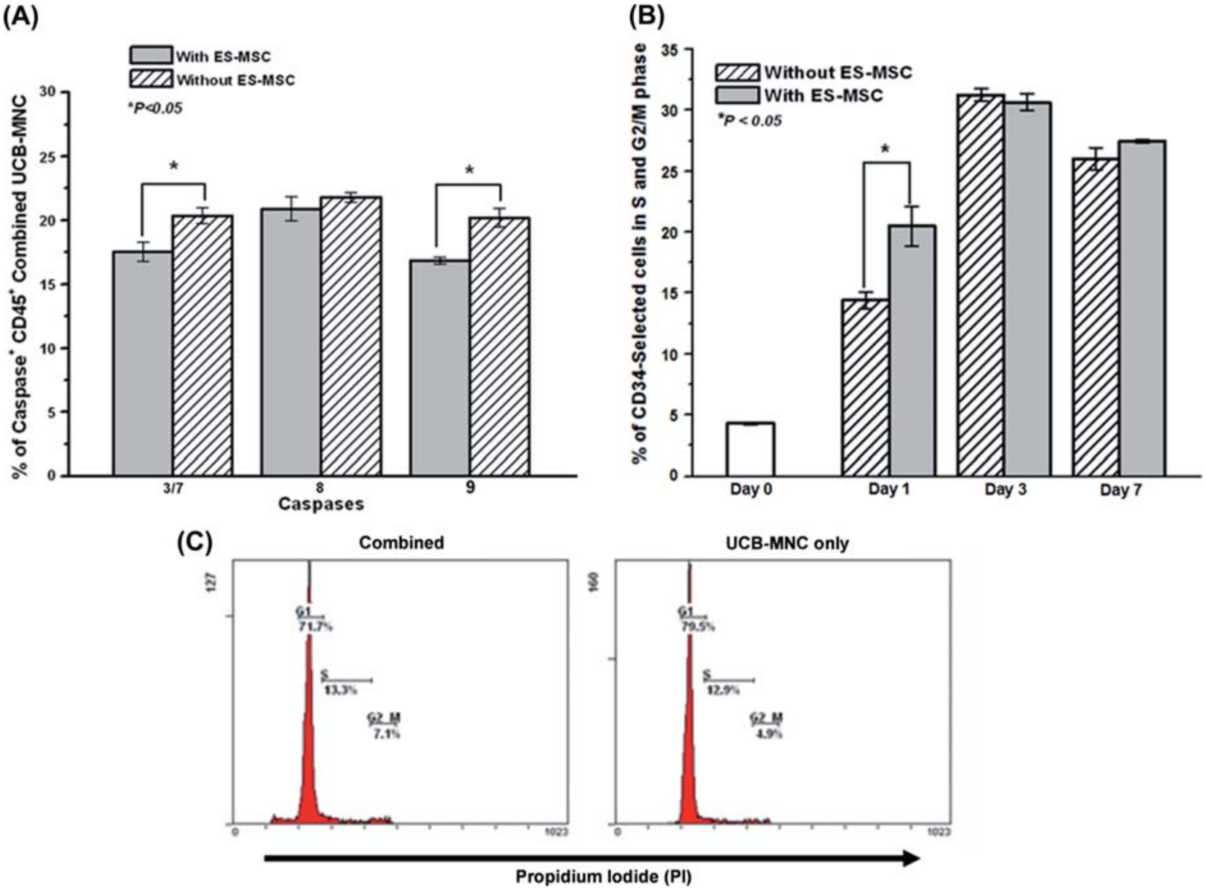

Supplementary Figure 2. Investigation of caspase activation and cell cycle analysis of the ES-MSC co-cultured UCB-MNC. (A) Activation of caspase 3/7, 8 and 9 (FLICA reagent) in combined CD45^{+} UCB-MNC on Day 3 (*represents $p < 0.05$). (B) Cell cycle analysis of CD34-selected cell population in the co-culture system. The DNA contents of the cell samples were stained with propidium iodide (PI) and analyzed by flow cytometry (*represents $p < 0.05$). (C) Representative flow cytometer plots showing the cell cycle analysis of the ES-MSC co-cultured (combined fraction) and non-co-cultured CD34 selected UCB-MNC. Data represent mean ± S.E.M. from three independent experiments.

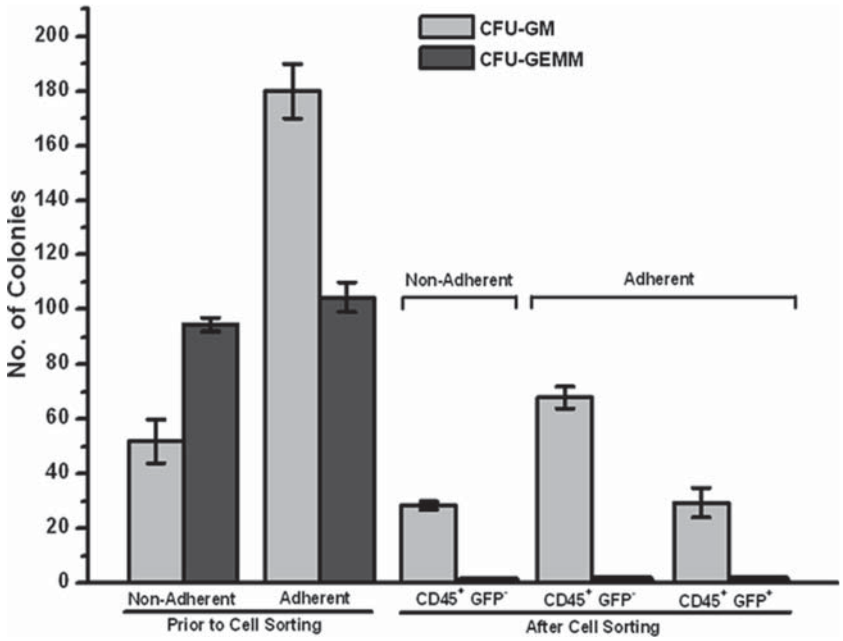

Supplementary Figure 3. CFU-GM and CFU-GEMM assay of the adherent $CD45^{+}$ GFP^{+} UCB-MNC. Fluorescence activated cell sorting (FACS) had a detrimental effect on the primitive colony forming cells. There is a complete depletion of the primitive CFU-GEMM colonies in the sorted group. CFU-GEMM is present in the control pre-sorted samples. Data represent mean $\pm$ S.E.M. from three independent experiments.
